# An E3 ubiquitin ligase localization screen uncovers DTX2 as a novel ADP-ribosylation-dependent regulator of DNA double-strand break repair

**DOI:** 10.1016/j.jbc.2024.107545

**Published:** 2024-07-09

**Authors:** Billel Djerir, Isabelle Marois, Jean-Christophe Dubois, Steven Findlay, Théo Morin, Issam Senoussi, Laurent Cappadocia, Alexandre Orthwein, Alexandre Maréchal

**Affiliations:** 1 Faculty of Sciences, Department of Biology, Université de Sherbrooke, Sherbrooke, Quebec, Canada; 2 Cancer Research Institute of the Université de Sherbrooke, Sherbrooke, Quebec, Canada; 3 Lady Davis Institute for Medical Research, Segal Cancer Centre, Jewish General Hospital, Montréal, Quebec, Canada; 4 Faculty of Sciences, Department of Chemistry, Université du Québec à Montréal, Montréal, Quebec, Canada; 5 Department of Radiation Oncology, Emory University School of Medicine, Atlanta, Georgia, USA

**Keywords:** DNA damage response, double-strand break repair, homologous recombination, ADP-ribosylation, ubiquitylation, PARP1, DELTEX family E3 ubiquitin ligases

## Abstract

DNA double-strand breaks (DSBs) elicit an elaborate response to signal damage and trigger repair *via* two major pathways: nonhomologous end-joining (NHEJ), which functions throughout the interphase, and homologous recombination (HR), restricted to S/G2 phases. The DNA damage response relies, on post-translational modifications of nuclear factors to coordinate the mending of breaks. Ubiquitylation of histones and chromatin-associated factors regulates DSB repair and numerous E3 ubiquitin ligases are involved in this process. Despite significant progress, our understanding of ubiquitin-mediated DNA damage response regulation remains incomplete. Here, we have performed a localization screen to identify RING/U-box E3 ligases involved in genome maintenance. Our approach uncovered 7 novel E3 ligases that are recruited to microirradiation stripes, suggesting potential roles in DNA damage signaling and repair. Among these factors, the DELTEX family E3 ligase DTX2 is rapidly mobilized to lesions in a poly ADP-ribosylation-dependent manner. DTX2 is recruited and retained at DSBs *via* its WWE and DELTEX conserved C-terminal domains. In cells, both domains are required for optimal binding to mono and poly ADP-ribosylated proteins with WWEs playing a prominent role in this process. Supporting its involvement in DSB repair, DTX2 depletion decreases HR efficiency and moderately enhances NHEJ. Furthermore, DTX2 depletion impeded BRCA1 foci formation and increased 53BP1 accumulation at DSBs, suggesting a fine-tuning role for this E3 ligase in repair pathway choice. Finally, DTX2 depletion sensitized cancer cells to X-rays and PARP inhibition and these susceptibilities could be rescued by DTX2 reexpression. Altogether, our work identifies DTX2 as a novel ADP-ribosylation-dependent regulator of HR-mediated DSB repair.

Maintenance of genome stability is central to cell homeostasis, organismal development, and reproduction (1). Therefore, all living organisms rely on complex signaling pathways to prevent, detect, signal, and repair the myriad of DNA lesions that threaten the integrity of their genetic material on a daily basis (2, 3). DNA double-strand breaks (DSBs) are highly deleterious lesions that can lead to genome rearrangements and/or rapid loss of essential genetic information if cells undergo division without repairing their chromosomes.

Two major DNA repair pathways are shared by all organisms to fix these breaks: nonhomologous end-joining (NHEJ), which in eukaryotes functions throughout interphase, and homologous recombination (HR) which is constrained to the S and G2 phases of the cell cycle (4). While mostly error-free, NHEJ repair may result in short indels caused by the activity of nucleases and polymerases at DNA ends (5). In contrast, HR is a conservative pathway that carries out recombination, ideally between sister chromatids, to promote seamless repair (6). The choice between these two pathways and their subsequent enactment is a highly coordinated process that requires the action of multiple DNA damage response (DDR) factors. Studies over the last two decades have shown that repair pathway choice is controlled by the opposing activities of 53BP1 and BRCA1/BARD1, a key suppressor complex. 53BP1 and its partners RIF1, REV7, and the Shieldin complex promote NHEJ, while the BRCA1/BARD1 complex, intervenes at multiple steps of break repair to promote HR (7, 8, 9, 10, 11, 12, 13, 14, 15, 16, 17, 18, 19, 20, 21, 22, 23, 24, 25, 26, 27).

The recruitment, exchange, activation, and eventual ejection and degradation of chromatin-associated histones and DNA repair factors at DSBs is heavily regulated by post-translational modifications (PTMs) which include ADP-ribosylation, phosphorylation, ubiquitylation, and SUMOylation (28, 29, 30). Frequent crosstalk between these PTMs is observed, with a common theme being that writers for specific modifications are often regulated by prior PTMs on their substrates (31). An interesting example of this is ADP-ribosylation (ADPr)-dependent ubiquitylation which, in recent years, has been increasingly recognized as an important component of the DDR (32). Protein ADPr is rapidly induced in response to DNA damage and is primarily mediated at DNA breaks by the ADP-ribosyl transferases PARP1, PARP2, and PARP3 (33, 34). Poly (ADP-ribose) polymerases (PARPs) catalyze the NAD^+^-dependent addition of poly- or mono-ADP ribose (ADPr, PARylation, or MARylation) molecules onto serine, glutamate, and aspartate residues of target proteins and were also recently shown to deposit ADPr onto nucleic acids (35, 36, 37, 38). MAR/PARylation is countered by specific glycohydrolases which include poly (ADP-ribose) glycohydrolase (PARG), TARG1, and ARH3 to control the ADPr chain length and duration of ADP ribosylation (39). The sheer abundance of PARP1 in the nucleus and its almost immediate accumulation at DNA breaks make it an important contributor but also an obstacle to proper DNA repair and genome stability (40, 41). The latter situation can be encouraged by PARP inhibitors (PARPis) which can trap PARP1/2 onto DNA, creating lesions that efficiently kill HR-defective cells through still debated mechanisms (42, 43, 44, 45). Four different PARP1/2 inhibitors including olaparib/Lynparza have been approved for clinical use in breast, ovarian, and prostate cancers. Understanding the determinants of PARPi sensitivity is thus an important endeavor (46, 47). Massive MAR/PARylation at DSBs has been proposed to relax chromatin, tether specific ADPr-reading repair factors and more recently to regulate the architecture of DNA-PARP1 condensates during end-joining and repair (48, 49, 50).

Many E3 ubiquitin ligases harbor ADPr-binding domains and have been shown to specifically ubiquitylate PARylated or MARylated proteins (32). The founding member of this group of ubiquitin ligases is the PAR-dependent E3 RNF146/Iduna which binds to PAR chains *via* its WWE domains and is allosterically stimulated by ADPr to ubiquitylate its substrates (31, 51, 52, 53). Among other E3 ligases that possess WWE domains are the HECT-type E3s TRIP12 and HUWE1 which also have demonstrated roles in the DDR and the DELTEX RING E3 family members DTX1, DTX2, and DTX4 (54, 55, 56). Recent proteomics analyses have suggested that DTX2 can bind to PARylated DDR factors and promote their ubiquitylation *in vivo* but whether DTX2 is required for genome maintenance and DNA repair has not yet been investigated (57). Here, we have performed a localization screen to identify novel E3 ubiquitin ligases involved in genome maintenance. We identified seven new RING E3 ubiquitin ligases that are recruited to DNA lesions and determined that four of them, including DTX2, accumulate on damaged DNA in a PARP-dependent manner. We find that DTX2 recruitment to DNA breaks is primarily mediated by its tandem WWE domains with additional contribution from its characteristic C-terminal DELTEX and RING domains. DTX2 depletion impedes BRCA1 foci formation and decreases the efficiency of HR repair. In agreement with these findings, DTX2-depleted cells are sensitive to X-ray irradiation and PARP inhibition, formally demonstrating the functional relevance of DTX2 in DNA repair and cell tolerance to genotoxic stress.

## Results

### A screen for E3 ubiquitin ligases involved in DNA repair

**Figure 1:**
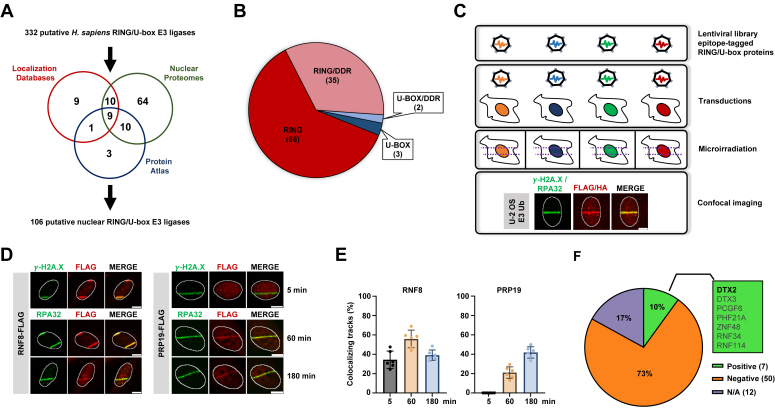
**A localization screen for human RING/U-box E3 ubiquitin ligases recruited to DNA damage.***A*, subcellular localization prediction for human RING/U-box E3 ubiquitin ligases. *B*, pie-chart annotation of the 106 putatively nuclear RING/U-box E3 ligases. Thirty-seven nuclear E3 ligases are already described as involved in the DNA damage response in the literature. The 69 (66 RING/3 U-box) remaining candidates were selected for a microirradiation localization screen. *C*–*E*, screen schematic and validation. U-2 OS cells were individually transduced with lentiviruses to express FLAG- or HA-tagged E3 ubiquitin ligases candidates. Forty-eight hours post selection, cells were microirradiated and immunofluorescence staining was performed using (FLAG/HA) and γ-H2A.X as early (5 min) or RPA32 as late (60/180 min) DNA damage markers. RNF8 and PRP19 were used as positive controls for screen optimization. Data represent the mean % of colocalizing E3 ligase and DNA damage marker tracks ± SD (each data point represents an independent biological replicate (n = 6)). *F*, pie-chart of screen results. Out of 69 candidates, seven were found to accumulate at microirradiation stripes, whereas 50 were unresponsive. Twelve candidates could not be tested for technical reasons. The scale bar represents 10 μm. HA, hemagglutinin; RPA32, replication protein A 32.

**Figure 2:**
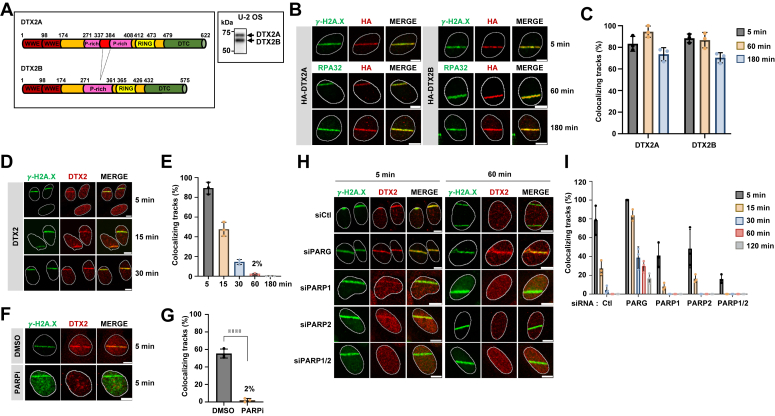
**DTX2 localizes to microirradiation stripes in a PARP-dependent manner.***A*, schematic representation of the 2 DTX2 isoforms and immunoblot of U-2 OS total cell extracts. The protein region corresponding to exon 4 is presented in *red* and is absent from DTX2B. *B* and *C*, DTX2A/B are recruited to microirradiation stripes. U-2 OS cells were individually transduced with lentiviruses encoding HA-tagged DTX2A/B. Subsequently, 48 h post selection, cells were microirradiated, and IF staining was achieved for HA and γ-H2A.X as early (5 min) or RPA32 as late (60/180 min) DNA damage markers. Quantification of microirradiated cells with DTX2A/B accumulation at positive tracks. Data represent the mean % of cells with DTX2A/B/γ-H2A.X or RPA32-colocalizing stripes ± SD (n = 3 biological replicates). *D* and *E*, kinetics of endogenous DTX2 recruitment at different time points (min) to microirradiation stripes ± SD (n = 3 biological replicates) (antibody validation in Fig. S2, *A*–*C*). *F* and *G*, PARP inhibition abrogates endogenous DTX2 recruitment to DNA damage. Cells were treated with vehicle (DMSO) or 5 μM PARPi (AZD-2281, olaparib) for 30 min prior to performing microirradiation and IF as in (*B*). Data represent the mean % of cells with endo DTX2/γ-H2A.X colocalizing stripes ± SD (n = 4 biological replicates). Statistical significance was established using an unpaired *t* test (*p* < 0.0001 (∗∗∗∗)). *H* and *I* influence of PARylation on DTX2 recruitment to microirradiation stripes. U-2 OS cells were transfected with siRNAs targeting PARG, PARP1, PARP2 or both and 48 h later, microirradiation and IF was performed. Data represent the mean % of cells with endo DTX2/γ-H2A.X colocalizing stripes ± SD (n = 3 biological replicates). In the bar graphs, each data point represents an independent biological replicate. The scale bar represents 10 μm. IF, immunofluorescence; PARG, poly(ADP-ribose) glycohydrolase; DMSO, dimethyl sulfoxide; PARP, poly (ADP-ribose) polymerase; PARPi, PARP inhibitor; RPA, replication protein A.

To uncover novel E3 ubiquitin ligases involved in the DDR, we surveyed online databases to define the nuclear RING/U-box E3 ubiquitin ligase landscape of human cells (Fig. 1*A* and Table S1). We first extracted all proteins annotated as nuclear from four databases: (1) The Human Protein Atlas (58), (2) UniProt (59), (3) LocDB (60) and (4) Gene Ontology (61). These putative nuclear proteomes were compared with a combination of two exhaustive lists of predicted human ubiquitin ligases (62, 63). RING/U-box E3 ligases that were annotated as nuclear in all databases were included as likely nuclear. We also interrogated two high quality mass spectrometry analyses of nuclear proteomes from *Xenopus laevis* and human cells (64, 65). Finally, we took advantage of a large-scale effort to map the subcellular localization of human proteins using specific antibodies. Proteins annotated as nuclear by immunofluorescence (IF) with validated antibodies in all cell types examined were included (58). Altogether, out of the 332 predicted RING/U-box E3 ubiquitin ligases, 106 were considered as likely nuclear proteins (Table S1). Of that number, 37 already had strong experimental evidence supporting their recruitment to damaged DNA and 12 were unavailable as full-length complementary DNAs (cDNAs) or too large for successful lentiviral packaging (Fig. 1, *B* and *F* and Tables S1 and S3). Coding sequences of the remaining 57 E3 ligases were cloned into lentiviral expression vectors in frame with FLAG or hemagglutinin (HA) epitope tags and individually transduced into U-2 OS cells (Fig. 1*C*). Microirradiation was then performed to preferentially induce DSBs (66). RNF8 and PRP19, which are respectively recruited rapidly onto chromatin after DSB induction or more slowly onto replication protein A (RPA)-ssDNA as DSB resection occurs were used as positive controls (Fig. 1, *D* and *E*) (67, 68, 69, 70, 71). Phosphorylated H2A.X (γ-H2A.X) and the RPA 32 kDa subunit (RPA32) were used for colabeling as DNA damage markers for early (5 min) and late (60 and 180 min) microirradiation time points, respectively (Fig. 1, *D* and *E*). Out of all 57 tested candidates, 50 were well-expressed but did not form detectable stripes. We also identified seven novel E3 ligases that formed clear stripes in microirradiated nuclei: DTX2, DTX3, PCGF6, PHF21A, ZNF48, RNF34, and RNF114 (Figs. 1*F*, S1, *A*–*C* and Table S3). Here, we chose to concentrate our characterization efforts on the DELTEX family protein, DTX2, as its recruitment was the most profound and striking among all candidates (Figs. 2, *A* and *B* and S1, *A*–*C*).

### DTX2 is recruited to DSBs in a PARylation-dependent manner

DTX2 is expressed as two alternatively spliced isoforms in human cells, DTX2A and B, with the latter lacking the fourth out of nine exons which encodes part of a proline-rich domain (Fig. 2*A* and (72)). HA-tagged versions of both isoforms were rapidly recruited to stripes and remained abundant over 3 h post microirradiation (Fig. 2, *B* and *C*). Endogenous DTX2 was also found to be recruited quickly but remained detectable only up to 60 min post damage in some cells (Fig. 2, *D* and *E*). The faster clearance time of endogenous DTX2 compared to HA-DTX2A/B suggests that overexpression might promote retention of DTX2 at DSBs. Importantly, knockdown (KD) of DTX2 using two independent siRNAs led to an almost complete abrogation of DTX2 stripes supporting the specificity of the antibody Fig. S2, *A*–*C*).

Both isoforms of DTX2 harbor tandem WWE domains at their N termini (Fig. 2*A*). WWE domains are known interactors of PAR chains found in multiple proteins including E3 ubiquitin ligases and PARPs (51, 73, 74). DTX2 was also recently found to ubiquitylate PARylated proteins, ADP-ribosylate ubiquitin and to ubiquitylate PAR on proteins and nucleic acids *via* its DELTEX C-terminal/RING domain module (57, 75, 76, 77). To test whether DTX2 recruitment to DSBs was PARylation-dependent, cells were pretreated with PARP1/2 inhibitor olaparib (AZD-2281/Lynparza) prior to microirradiation. Strikingly, HA-tagged and endogenous DTX2 localization to microirradiation stripes was almost completely abrogated by PARP1/2 inhibition (Figs. 2, *F* and *G* and S2, *D* and *E*). Under the same conditions, recruitment of RNF8 remained unaltered in PARPi-treated cells in agreement with its MDC1 phosphorylation-dependent recruitment (Fig. 2, *F* and *G*) (67, 70). Conversely, PAR chain stabilization through PARG depletion greatly increased the initial recruitment and lengthened the retention time of endogenous DTX2 at stripes (Figs. 2, *H* and *I* and S2*J*). Depletion of PARP1 by siRNA (KD) also decreased but did not completely impede DTX2 recruitment suggesting that other PARP enzymes attract DTX2 to DSBs. Codepletion of PARP2, also known to be inhibited by olaparib, led to an almost complete disappearance of DTX2 at microirradiated stripes (Figs. 2, *H* and *I* and S2*J*). Interestingly, the recruitment of three other E3 ligases identified in the screen, the DELTEX family protein DTX3, LSD1-coREST component PHF21A and RNF114, was also abrogated by prior treatment of cells with PARPi, suggesting that PARylation is important for their accumulation and retention at DNA lesions (Fig. S2, *H* and *I*).

### The WWE and DTC domains mediate the recruitment of DTX2 to DSBs

**Figure 3:**
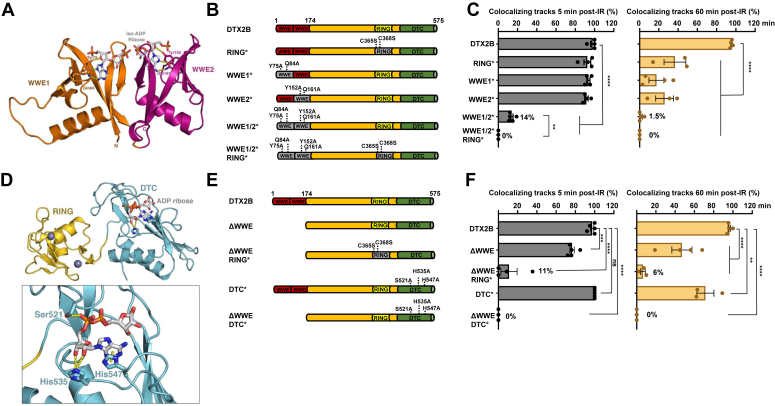
**The WWE1/2 and DTC domains of DTX2 mediate its recruitment to microirradiation stripes.***A*, structural model of the tandem DTX2 WWE domains in complex with 2 iso-ADP-ribose molecules based on X-ray crystallography data from the RNF146 WWE bound to iso-ADPr complex (Wang *et al.* G&D 2012). Key mutated residues that interact with iso-ADP-ribose are highlighted. *B* and *C*, the WWE1/2 domains are essential for DTX2 recruitment at microirradiation stripes. U-2 OS cells were individually transduced with lentiviruses encoding HA-tagged DTX2B or the indicated mutants. Forty-eight hours post selection, cells were microirradiated and immunofluorescence staining for HA and γ-H2A.X as a DNA damage marker. Quantification of micro-irradiated cells with DTX2 constructs accumulation at γ-H2A.X-positive stripes. *D*, structural model of the RING-DTC domain of DTX2 in complex with ADP-ribose based on X-ray crystallography data (Ahmed *et al.* Sci. Adv. 2020). Key mutated residues that interact with ADP-ribose are highlighted. *E* and *F*, the DTC domain mediates DTX2 recruitment to microirradiation stripes upon WWE1/2 truncation. The indicated HA-tagged DTX2 mutants were transduced into U-2 OS cells and microirradiation and immunofluorescence were performed. Data represent the mean % of cells with colocalizing DTX2/γ-H2A.X ± SEM (n ≥ 3 biological replicates). In the bar graphs, each data point represents an independent biological replicate. Statistical significance was established using One-Way ANOVA followed by Dunnett's multiple comparison test (∗∗ *p* < 0.01, ∗∗∗ *p* < 0.001, ∗∗∗∗ *p* < 0.0001). ADPr, ADP-ribosylation; DTC, DELTEX conserved C-terminal; HA, hemagglutinin.

To better characterize the recruitment of DTX2 to DNA damage, two mutations were introduced in each of the WWE N-terminal domains (WWE1∗:Y75A/Q84A and WWE2∗:Y152A/Q161A) at residues equivalent to those previously found to interact with iso-ADPr in the WWE domain of RNF146 (Fig. 3, *A* and *B*) (51). A quadruple WWE1/2∗ mutant, expected to be unable to associate with iso-ADPr *via* its WWE domains was also created. Mutation within single WWE or RING domains (C365S/C368S) did not affect early recruitment of DTX2 to microirradiation stripes but led to decreased retention of these proteins at later time points (Fig. 3, *B* and *C*). Remarkably, mutation of both WWE domains strongly abrogated the formation of DTX2B microirradiation stripes without otherwise affecting its subcellular distribution pattern (Figs. 3, *B* and *C* and S3, *A*–*C*).

In contrast to the WWE1/2∗ mutant, complete truncation of the 2 WWE domains (ΔWWE) allowed for diminished but readily detectable recruitment of DTX2B at microirradiation stripes, suggesting that removal of the DTX2B N terminus allows another domain to tether the protein to sites of damage (Fig. 3, *E* and *F*). The DELTEX conserved C-terminal (DTC) domain was recently shown to form a functional module with the RING domain and promote ADPr-dependent ubiquitylation of substrates (57). To determine whether the DTC is responsible for the recruitment that subsists in the absence of the tandem WWE domains, missense mutations were introduced in three residues (S521A/H535A/H547A) that mediate the DTC-ADPr association (57) (Figs. 3, *D* and *E* and S3, *A*–*C*). In the presence of functional WWE domains, the DTC mutation decreased the intensity of the stripes and significantly impaired the retention of DTX2 60 min post irradiation (Figs. 3*F* and S3*B*). Furthermore, DTC mutation completely abrogated the recruitment of the ΔWWE construct, indicating that this domain tethers DTX2 to DNA lesions in the absence of its WWE domains (Figs. 3*F* and S3*B*). Interestingly, we also noted that mutation of the RING domain (C365A/C368S) decreased the intensity and retention of full-length DTX2B at stripes and that its comutation with the WWE domains abrogated early and late recruitment, implicating the E3 ligase motif of DTX2 in its mobilization to DNA lesions (Figs. 3, *B*, *C*, *E* and *F* and S3, *A*–*C*).

**Figure 4:**
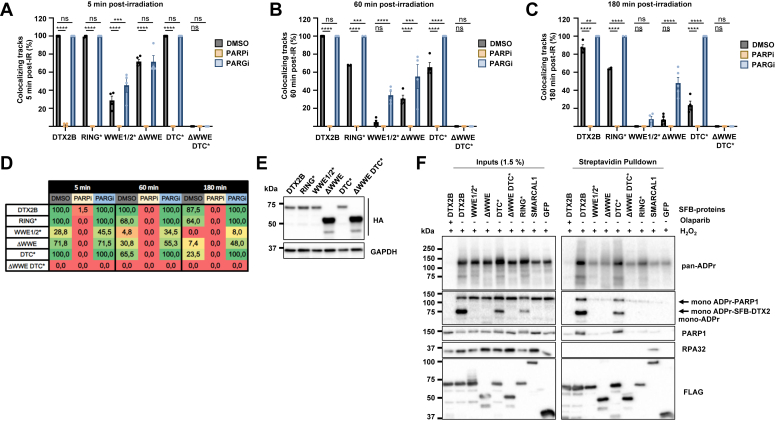
**The WWE and DTC domains of DTX2 associate with ADP-ribosylated proteins *in vivo*.***A*–*C*, U-2 OS cells were individually transduced with lentiviruses encoding HA-tagged DTX2B or the indicated mutants. Forty-eight hours post selection, cells were microirradiated and IF staining for HA and γ-H2A.X was performed on fixed cells 5, 60, and 180 min post microirradiation. Data represent the mean % of cells with colocalizing HA/γ-H2A.X ± SEM (n ≥ 3 biological replicates). In the bar graphs, each data point represents an independent biological replicate. Statistical significance was established using two-way ANOVA followed by Dunnett's multiple comparison test (∗∗ *p* < 0.01, ∗∗∗ *p* < 0.001, ∗∗∗∗ *p* < 0.0001. *D*, summary table of the microirradiation experiments presented in (*A*–*C*). Numbers represent the average value of at least six microirradiated fields. *E*, immunoblot validation of HA-DTX2 mutant expression (*F*) HEK293 cells were individually transfected with plasmids encoding SFB-tagged DTX2B constructs or SMARCAL1 and EGFP controls. Forty-eight hours later, cells were treated with H_2_O_2_ in the presence or absence of olaparib, lysed, and streptavidin pull-down was performed. DTX2B and its interactors were detected by immunoblotting. DTC, DELTEX conserved C-terminal; HA, hemagglutinin; IF, immunofluorescence; SFB, S protein-FLAG-streptavidin binding peptide.

To formally demonstrate that the residual recruitment of WWE1/2∗, ΔWWE, and DTC∗ constructs was still dependent on mono-ADP or poly-ADP-ribosylation, we performed microirradiations in cells pretreated with PARPi or PARGi (Fig. 4, *A*–*E*). The absence of stripes in PARPi-treated cells confirmed that recruitment of both WT and mutant DTX2 required PARP1/2 activity. Interestingly, late recruitment (60–180 min post irradiation) of WWE1/2∗ and ΔWWE and DTC∗ mutants was enhanced by PARGi suggesting that increased stabilization of PAR chains at DNA lesions can partially palliate for the absence of WWE or DTC ADP-ribose-binding activity. PARGi could not increase the recruitment of ΔWWE-DTC∗ indicating that this mutant is completely unable to interact with ADP-ribosylated moieties induced by DNA damage. Altogether, our data show that the WWE domains are the principal mediators of DTX2 ADP-ribosylation-dependent recruitment at DNA lesions whereas the DTC and RING domains play a less prominent role in this process.

### DTX2 interacts with MAR- and PARylated proteins in cells mainly *via* its WWE domains

These data and prior work from the Huang and Ahel labs suggest that DTX2 interacts with and may ubiquitylate PARylated proteins and/or nucleic acids in response to DNA damage *via* its WWE, RING, and DTC domains (57, 76). To test the domain requirements of DTX2 for interaction with ADP-ribosylated proteins, we expressed SFB-tagged DTX2 constructs in cells treated with H_2_O_2_ to induce PARP activation and performed streptavidin pull-downs. WT and DTC∗ constructs readily associated with PARylated proteins and PARP1. Moreover, the association of DTX2 with these factors was completely abrogated by pretreatment of cells with PARPi indicating that ADPr was required for these interactions (Fig. 4*F*). The ΔWWE DTC∗ mutant was completely unable to associate with PARylated proteins but interestingly, the ΔWWE and WWE1/2∗ constructs retained a very mild association with PARylated factors compared with controls (GFP and SMARCAL1). This weakened but still detectable association of ΔWWE and WWE1/2∗ with PARylated proteins may explain their residual recruitment to microirradiation stripes (Fig. 4, *A*–*F*). We note that the WWE1/2∗ interaction with PARylated proteins is only visible upon prolonged exposure of the blots (Fig. S4*A*). We also observed a mild decrease in the association of the DTC∗ mutant with MARylated proteins detected with a specific mono-ADPr antibody (PARP1 and SFB-DTX2) (78). This defect was more pronounced for the RING∗ construct which was completely unable to interact with PARP1 and had decreased association with PARylated proteins. Although DTX2 was previously suggested to interact with the RPA complex *in vivo*, we were unable to detect an association between SFB-DTX2 and RPA32 in H_2_O_2_-treated cells whereas SMARCAL1, a known interactor of RPA showed strong association with the complex but no interaction above background with ADP-ribosylated factors (Fig. 4*F* and (57)). However, as reported, a robust interaction with PARP1 was detected for both full length and DTC∗ DTX2B. Altogether, our data indicates that intact WWE, DTC and RING domains are required for optimal association with PARylated and/or MARylated factors and that the WWE domains are the main mediators of these interactions.

### DTX2 influences DSB repair efficiency

**Figure 5:**
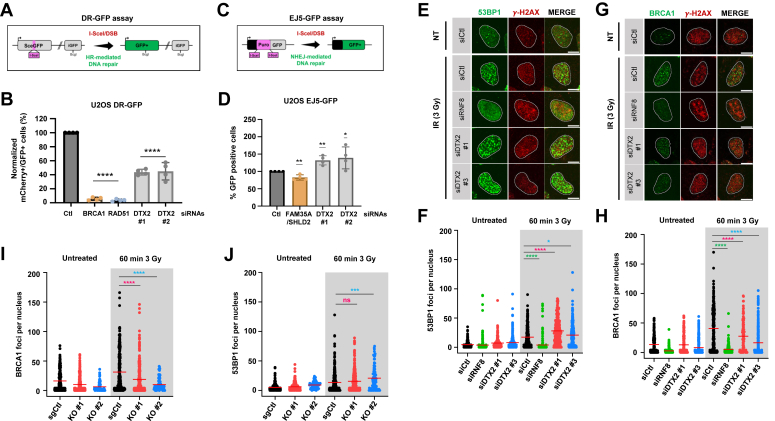
**DTX2 influences double-strand break repair.***A* and *C*, schematic representation of the DR-GFP and EJ5-GFP homologous recombination and nonhomologous end-joining fluorescent reporter systems. *B*, U-2 OS DR-GFP cells were transfected with the indicated siRNAs and subsequently cotransfected with mCherry and I-Sce-I-expressing plasmids. Homologous recombination efficiency is measured as the % of GFP- and mCherry-positive cells and HR efficiency was normalized to control cells. The graph represents the mean −/+ standard deviation obtained from four biological replicates (data points on the bar graph). *D*, U-2 OS EJ5-GFP cells were transfected with the indicated siRNAs. Twenty-four hours post transfection, cells were transfected with the I-SceI expression plasmid, and the GFP+ population was analyzed 48 h post plasmid transfection. The percentage of GFP+ cells was determined and subsequently normalized to control cells. Data are presented as the mean −/+ standard deviation from four biological replicates (data points on the bar graph). *E* and *F*, U-2 OS cells transfected with the indicated siRNAs and 48 h later were irradiated or not with 3 Gy of X-rays and processed for 53BP1 or (*G* and *H*) and BRCA1 immunofluorescence. The scale bar represents 10 μm. *I* and *J* sgCtl or DTX2 KO U-2 OS cells were irradiated or not with 3 Gy of X-rays and 1 h later processed for 53BP1 and BRCA1 immunofluorescence. Significance for all graphs was determined by one-way ANOVA followed by a Dunnett’s test. (∗ *p* < 0.05, ∗∗ *p* < 0.01, ∗∗∗ *p* < 0.001, ∗∗∗∗ *p* < 0.0001). HR, homologous recombination.

There are six human E3 ubiquitin ligases that contain a WWE PAR-binding domain: RNF146/Iduna, TRIP12, HUWE1, DTX1, DTX2, and DTX4 (55). Among these, RNF146, TRIP12, and HUWE1 were previously shown to participate in the regulation of DSB repair and promote genome stability (52, 79, 80, 81). To determine whether DTX2 is directly involved in DSB repair, we first tested its role in two canonical DSB repair pathways, HR and NHEJ, using the well-characterized DR-GFP and EJ5-GFP reporter assays, respectively (82, 83). Depletion of DTX2 using two independent siRNAs led to a significant decrease of approximately 50% in HR efficiency suggesting an active role in this repair pathway (Figs. 5, *A* and *B* and S5*A*). Conversely, depletion of DTX2 led to a mild increase in NHEJ efficiency (Fig. 5, *C* and *D*). We then monitored the accrual of two key regulators of these pathways, 53BP1 and BRCA1, to DSBs induced by X-ray irradiation. KD of DTX2 using two siRNAs slightly increased X-ray-induced 53BP1 foci numbers (Figs. 5, *E* and *F* and S5*B*). Conversely, BRCA1 foci numbers were significantly decreased by DTX2 KD in agreement with defective HR repair (Fig. 5, *G* and *H*). Similar results were also obtained in two independently derived DTX2 KO cell lines (Figs. 5, *I* and *J* and S5, *C* and *D*). Importantly, DTX2 KD or KO did not lead to significant alterations in cell cycle distribution indicating that the effects on HR and NHEJ were not due to a decrease in the fraction of S/G2 cells (Fig. 5, *E* and *F*).

### DTX2 promotes tolerance to X-rays and olaparib

**Figure 6:**
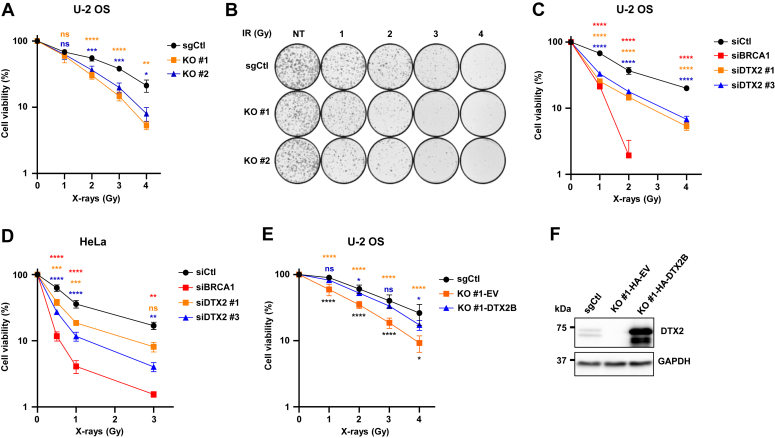
**DTX2 promotes resistance to X-rays.***A* and *B*, sgCtl or DTX2 KO U-2 OS cell lines were exposed to the indicated doses of X-ray irradiation and colony formation assays were performed over 12 days and representative colonies are shown. *C* and *D*, U-2 OS or HeLa cells were transfected with the indicated siRNAs and 48 h later exposed to the indicated doses of X-rays. Colony formation assays were done over 12 days *E*, sgCtl or DTX2 KO cells stably transduced with empty vector or HA-DTX2B carrying vector were exposed to the indicated X-ray doses and colony formation assays were done over 12 days *F*, immunoblot validation of DTX2 expression levels. For all CFA datasets, ≥ 3 biological replicates were done, and data represent the mean −/+ standard error of the mean. Statistical significance was computed using 2-way ANOVA with Tukey’s multiple comparisons (∗ *p* < 0.05, ∗∗∗ *p* < 0.001, ∗∗∗∗ *p* < 0.0001). HA, hemagglutinin.

To further document the functional importance of DTX2 in the outcome of DSB repair, we performed clonogenic assays. In agreement with a role for DTX2 in break repair, DTX2 KO U-2 OS cell lines were significantly more sensitive to X-ray exposure than sgCtl cells (Fig. 6, *A* and *B*). Moreover, siRNA-mediated depletion of DTX2 in both U-2 OS and HeLa cells also conferred increased sensitivity to irradiation (Fig. 6, *C* and *D*). Similarly to the milder impact of DTX2 depletion on HR efficiency compared with BRCA1 KD, we note that depletion of DTX2 was less impactful on X-ray sensitivity compared with BRCA1, suggesting that DTX2 may play a more accessory role in DSB repair than core HR factors (Figs. 5*B* and 6, *C* and *D*). Importantly, stable transduction of full-length HA-tagged DTX2B significantly rescued the X-ray sensitivity of DTX2 KO cells, confirming that this phenotype is caused by the absence of DTX2 (Figs. 6, *E* and *F* and S6, *A* and *B*). Defects in HR also confer sensitivity to PARPi (84, 85). To test whether DTX2 is also involved in PARPi resistance, we performed DTX2 KD in U-2 OS, HeLa and RPE1-hTERT Δp53 cells and carried out colony formation (U-2 OS/HeLa) and viability assays (RPE1-hTERT) in the presence of increasing concentrations of olaparib (Fig. S6, *C*–*E*). In all three cell lines, resistance toward olaparib decreased upon DTX2 depletion. Moreover, U-2 OS DTX2 KO cells were also more susceptible to PARP inhibition, and this sensitivity could be rescued by reexpressing HA-tagged DTX2 (Fig. 6, *F* and *G*). Altogether, our data show that DTX2 is involved in DSB repair *via* HR and promotes resistance toward ionizing X-ray irradiation and PARP inhibition.

## Discussion

Here, we have performed a localization screen to identify novel E3 ubiquitin ligases from the RING and U-box families that function in genome stability maintenance. Our approach led to the identification of 7 RING E3 ligases that localize rapidly to microirradiated loci, suggesting potential roles in DNA damage signaling and repair (Figs. 1 and S1). Among these factors, at least four of them depend on PARP1/2 activity for their recruitment to DNA lesions (Figs. 3 and S2). These include PHF21A/BHC80, a PHD finger containing-chromatin reader whose mutation causes intellectual disabilities and craniofacial dysmorphisms including microcephalies (86, 87). Although the neurological defects related to *PHF21A* mutation have been ascribed to its role as part of the BRAF35 transcriptional repressor complex, some of the observed phenotypes may be related to a putative role in DNA repair, whether this is the case warrants further investigation (88). We also identified the multizinc finger protein RNF114/ZNF313 as a PARP-dependent E3 ligase recruited to DSBs. While this manuscript was in preparation, RNF114 was independently found to interact with mono-ADP-ribosylated proteins at sites of DNA damage, to promote NHEJ and regulate alternative lengthening of telomeres (89, 90). Consistent with these reports, we found that RNF114 recruitment to sites of damage depends on its second and third zinc fingers but also intriguingly on its C-terminal ubiquitin-interacting motif, perhaps suggesting dual recognition of ADP-ribose and ubiquitin (Fig. S7, *A*–*C*).

Most strikingly, our screen picked up 2 DELTEX family members, DTX2 and DTX3, as being recruited to DNA damage in a PARylation-dependent manner. In line with these results, DTX2 was recently identified both as a MAR- and PAR-binding factor in *ex vivo* nuclear proteomics analyses and shown to become MARylated and PARylated in response to genotoxic stress (91, 92, 93). We note that whereas endogenous DTX2 accrued rapidly to sites of damage and was virtually undetectable 1 h after microirradiation as expected from a PAR-binding protein, ectopically expressed DTX2 had a longer residency time (Fig. 2, *B*–*E*). We postulate that increased binding of DTX2 to ADPr chains may shield them from enzymatic degradation, thereby prolonging its recruitment to DNA lesions. Although DTX3 lacks obvious PAR-binding domains, we show here that it is also recruited to microirradiation stripes in a PARP1/2-dependent manner. One possibility may be that akin to DTX3L which also lacks WWE domains and relies on PARP9 to recognize PARylated proteins, DTX3 may associate with a partner to promote its recruitment to DNA lesions (94). As DELTEX E3s homodimerize and heterodimerize, DTX3 could potentially be recruited to DNA lesions *via* its association with another family member (72, 95, 96, 97). In support for the functional relevance of DELTEX dimerization, Notch signaling in drosophila requires homodimerization of the DELTEX homolog *via* its RING-H2 domain (95, 96, 97). More recently, oligomerization by DTX3L was also shown to be important for optimal ADPr binding (98, 99). Along these lines, we found that inactivating mutations within the DTX2 RING perturbed its recruitment to microirradiation stripes and its association with ADP-ribosylated factors (Figs. 3 and 4). AlphaFold multimer analyses further suggest that DTX2 can self-interact *via* its RING and DTC domains (Fig. S7, *A*–*D* and Table S4). This opens up the possibility that perturbation of homodimerization may impede DTX2 binding to MAR-/PARylated proteins and/or nucleic acids in response to DNA damage. Apart from the RING motif, our data also support a role for the DTC domain in the retention of DTX2 at microirradiation stripes and its association with MARylated PARP1 (Figs. 3 and 4).

Altogether, our data clearly point toward the WWE tandem domains as the most important determinants for ADP-ribosylated protein binding by DTX2 and its recruitment to DNA lesions (Figs. 3 and 4). Surprisingly, we also found that whereas mutation of WWE1/2∗ almost completes abrogates DTX2 recruitment to microirradiation stripes and interaction with MAR-/PARylated proteins, truncation of these domains led to much better accumulation of DTX2 at stripes and interaction with PARylated proteins compared with the WWE1/2∗ mutant. This is consistent with prior data showing that the DTX2 RING/DTC module is sufficient for ubiquitylation of PARylated targets *in vivo* and *in vitro.* The recruitment to sites of damage and residual interaction with PARylated factors of the ΔWWE mutant was fully dependent on a functional DTC, in agreement with its demonstrated ADPr affinity (57). These results have two potential implications: (1) in the complete absence of the WWE domains, the RING-DTC module can interact with PARylated substrates at sites of DNA damage and (2) when present but mutated, the WWE domains appear to restrain the ability of the RING-DTC to interact with PARylated proteins in cells, perhaps limiting the activity of DTX2 in the absence of DNA damage. Interestingly, although our initial bioinformatics analyses did not consider WWE domain-containing DTX1 and DTX4 as likely to be nuclear, both proteins are readily recruited to stripes when overexpressed in cells (Fig. S9, *A*–*C*). In support of these results, DTX4 present in HeLa nuclear extracts was also found to interact with mono-ADPr and poly-ADPr in pull-down experiments (91). Altogether, prior studies and the work presented here demonstrate that all five members of the DELTEX family (DTX1, DTX2, DTX3, DTX3L, and DTX4) can rapidly congregate onto DNA lesions and could potentially function as DNA damage signaling and repair regulators, roles which had already been demonstrated for DTX3L and now for DTX2 (94, 100). Whether endogenous DTX1 and DTX4 are also recruited to laser-induced DNA damage will be important to determine.

Prior research efforts have suggested roles for DTX2 in the DDR and we now provide strong functional evidence for its role in the maintenance of genome stability (57, 92). We show that DTX2 depletion decreases the efficiency of DSB repair *via* HR and concomitantly slightly increases NHEJ usage (Fig. 5, *A*–*D*). Impairment of HR was mirrored by a decrease in BRCA1 foci formation upon DTX2 KD or KO while a mild increase in 53BP1 accumulation at DSBs was noted and could potentially be linked to enhanced use of NHEJ. Overall, the impact of DTX2 depletion on BRCA1 and 53BP1 foci formation was relatively minor, in stark contrast to RNF8 KD which completely abrogated BRCA1 and 53BP1 X-ray-induced foci formation (Fig. 5, *E*–*J*). This suggests that DTX2 may work to fine-tune DSB repair or that it might be functionally redundant with other factors. We further found that KD or KO of DTX2 sensitized U-2 OS and HeLa cells to X-ray irradiation (Fig. 6, *A*–*F*). As expected for an HR regulator, DTX2 downregulation also led to marked sensitivity to olaparib in nontransformed (RPE1-hTERT Δp53) and cancerous (U-2 OS and HeLa) cell lines (Fig. S6, *A*–*G*) (43, 101). In agreement with these data, DTX2 was recently identified as one of the top PARPi-sensitizing hits in genome-wide CRISPR screens in two out of four examined BRCA1/2-proficient prostate cancer cell lines (22Rv1 and DU145) (102). A large-scale CRISPR-screen also revealed a mild sensitivity to olaparib upon DTX2 depletion in RPE1 Δp53 cells which we confirm here (103). In the longer term, understanding the specific contexts in which DTX2 inactivation may render cells sensitive to olaparib could be relevant to the clinical use of PARPi.

While our manuscript was in preparation, a preprint article describing the recruitment of DTX1 and DTX2 to DNA lesions and their function in DSB repair was deposited online (104). In this work, DTX1/2 recruitment to DSBs was shown to be PARylation-dependent and required its intact WWE tandem domains. Single guide RNA (sgRNA)-mediated DTX1/2 depletion also led to a marked ionizing radiation sensitivity in HeLa cells which could be rescued by WT but not WWE mutant DTX1/2, in accord with our own results (Figure 2, Figure 3, Figure 4 and 6). Furthermore, Kang *et al.* showed that ionizing radiation-induced BRCA1 and 53BP1 foci formation was perturbed in DTX1/2-depleted cells as were both HR and NHEJ pathways in contrast with our own results where DTX2 depletion led to impaired HR and BRCA1 foci. These discrepancies may be due to the use of different DTX2 depletion methods (siRNA *versus* sgRNA), cell lines (U-2 OS *versus* HeLa) and source of damage (X-rays *versus* γ-rays). Altogether, our data and that from other labs support a role for DTX2 as a novel regulator of DSB repair and an important mediator of irradiation and olaparib resistance.

While our study directly implicates DTX2 in genome maintenance for the first time, much remains to be determined in terms of the mechanistic underpinnings of its roles in DSB repair and more generally in the response to DNA damage. For instance, although we were unable to detect an association between DTX2 and RPA as previously suggested by proteomics, we did confirm a robust WWE domain-dependent DTX2/PARP1 interaction (Fig. 4*F* and (57)). Many E3 ubiquitin ligases have been shown to ubiquitylate PARP1 in response to damage and promote its proteasomal degradation, including RNF146/Iduna, TRIP12, CHFR, and more recently RNF114 (52, 55, 90, 105). Whether PARP1 is also targeted by DTX2 and other DELTEXs *in vivo* and what the extent of the functional redundancy/crosstalk is between the many ADPr-dependent E3 ligases that congregate at DNA damage sites will be important questions to tackle in future studies. In recent years, the DELTEX family has emerged as a particularly versatile group of ADPr-binding E3 ligases in terms of their substrate repertoire. DELTEX targets now include unmodified proteins and ADP-ribosylated proteins and nucleic acids (57, 72, 76, 100, 106, 107). Moreover, DELTEX E3s can also carry out ADPr of the ubiquitin C terminus, a function previously ascribed to the PARP9 partner of DTX3L (77, 108). Even more intriguingly, direct ubiquitylation of the 3′ ends of single- and double-stranded nucleic acids by the RING-DTC modules of DTX3L and to a lesser extent DTX3 has just been demonstrated *in vitro* (100, 109, 110). Determining what the specific substrates of DTX2 and other DELTEXs are during the DDR and how these ubiquitylation events govern repair pathway choice and genome stability is bound to be an exciting area for new discoveries.

## Experimental procedures

### Cell culture and treatments

Human U-2 OS, HeLa, and HEK293T cells were obtained from the American Type Culture Collection, U-2 OS DR-GFP/EJ5-GFP and RPE1-hTERT Δp53 cells were kindly provided by Jeremy Stark (Beckman Research Institute, City of Hope) and Daniel Durocher (Lunenfeld-Tanenbaum Research Institute, Toronto). Cell lines were grown in Dulbecco’s modified Eagle’s medium supplemented with 1% streptomycin/penicillin antibiotics (Wisent) and 10% fetal bovine serum (FBS, Gibco). Cells were grown at 37 °C in a 5% CO_2_ humidified atmosphere. Cells were regularly tested to ensure the absence of mycoplasma contamination. For treatments, olaparib (Selleck Chemicals), hydrogen peroxide (Sigma-Aldrich), and PARG inhibitor PDD00017273 (Sigma-Aldrich) were used as indicated in the corresponding figure legends.

### DNA constructs and cloning

Candidate ORFs expressing clones inserted into the lentiviral vector pReceiver-Lv242 in frame with a C-terminal FLAG epitope tag were obtained from Genecopoeia. For positive candidates, cDNAs were amplified by PCR and inserted by BP Gateway recombination into a pDONR221 entry vector with a STOP codon (Thermo Fisher Scientific). To generate mutants of DTX2B and RNF114, pDONR221-DTX2B and pDONR221-RNF114 were used as PCR templates for site-directed mutagenesis with appropriate oligonucleotides (Table S5). pDONR221 DTX2B-DTC∗ and pDONR221 DTX2B-ΔWWE-DTC∗ were synthesized by Gene Universal Inc cDNAs were transferred from pDONR221 into the lentiviral expression vector pEF1α-3xHA by LR Gateway recombination and validated by Sanger sequencing.

### Generation of cell lines

Individual lentiviruses were prepared in 6-well plates by cotransfection of candidate expression vectors and standard packaging plasmids (pMD2.G and psPAX2) into HEK293T cells. Viral supernatants were collected after 2 days, aliquoted in small volumes into 1.5 ml tubes, and stored at −80 °C. U-2 OS cells were infected with defrosted viruses by parallel transduction in 6-well plates, and transduced cell lines were selected in media supplemented with 1 μg/ml puromycin. In preparation for laser microirradiation, cell lines were plated on μ-Plate 96 Well Black ibiTreat (Ibidi).

### Laser microirradiation

Microirradiation was carried out as previously described (66). Cells were treated 15 min with 10 μg/ml Hoechst 33342 and rinsed twice with medium without phenol red. Before microirradiation, media were replaced with Dulbecco’s modified Eagle’s medium without phenol red. Microirradiation was performed using the Olympus FLUOVIEW FV3000 laser scanning confocal microscope equipped with a 405 nm laser line. Cells were then allowed to recover for a defined amount of time before fixation with PBS containing 3% paraformaldehyde and 2% sucrose and immunostaining.

### Immunoblotting

Total cell lysates were prepared using Laemmli buffer (120 mM Tris (pH 6.8), 12% glycerol, 3.67% SDS, 200 mM DTT, and bromophenol blue). Following resuspension, samples were boiled for 5 min at 95 °C and sonicated twice 10 s at 15% intensity on a Branson Sonicator (Branson 450 Digital Sonifier). Samples were then separated by SDS-PAGE and transferred onto 0.45 μm polyvinylidene fluoride membrane (Millipore). Membranes were blocked with 5% milk in Tris-buffered saline (TBS)/0.2% Tween-20 (TBS-T) and incubated with the appropriate primary antibodies (Table S5) followed by 3X TBS-T washes and incubation with the corresponding secondary antibodies (Table S5). Enhanced chemiluminescence reagent (GE HealthCare) was used for detection.

### Immunofluorescence

Cells were seeded onto coverslips and treated as described in the corresponding figure legends. Cells were washed twice with 1x PBS and prepermeabilized with ice-cold 1x PBS containing 0.25% Triton X-100 for 15 min. Cells were rinsed with 1x PBS and fixed with PBS containing 3% paraformaldehyde and 2% sucrose for 15 min at room temperature (RT). Cells were rinsed with 1x PBS and permeabilized with PBS containing 0.25% Triton X-100 for 15 min on ice. Cells were rinsed with 1x PBS and incubated with blocking buffer (3% bovine serum albumin, 0.05% Tween-20 in PBS) for at least 30 min at RT. Cells were incubated overnight at 4 °C in a humidified chamber with the first primary antibody diluted in the blocking buffer. Coverslips were rinsed with PBS-Tween 0.05% and incubated 1 h at 37 °C in a humidified chamber with the second primary antibody. Coverslips were rinsed with PBS-Tween 0.05% and incubated 1 h at 37 °C in a humidified chamber with secondary antibodies conjugated with Alexa Fluor 488 or Alexa Fluor 647 (Thermo Fisher Scientific). Samples were rinsed with PBS-Tween 0.05%, then incubated 5 min with PBS containing 4′,6-diamidino-2-phenylindole (1 μg/ml) and rinsed with PBS. Samples were mounted with ProLong Diamond Antifade Mountant (Thermo Fisher Scientific). Images were collected using a 40X objective lens on Olympus FLUOVIEW FV3000 laser scanning confocal microscope. Images were processed with Fiji and foci were automatically counted using CellProfiler (111, 112).

### RNA interference

Reverse transfection of siRNAs targeting DTX2, PARP1, PARP2, PARG, BRCA1, RNF8, RAD51, and FAM35A/SHLD2 were performed using Lipofectamine RNAiMAX (Thermo Fisher Scientific) according to the manufacturer’s instructions to transiently knockdown the indicated genes. The siRNAs sequences are provided in Table S5.

### Analysis of DTX2 structures

The model for the structure of DTX2 was obtained from the AlphaFold Database (accession Q86UW9). The position of iso-ADP-ribose molecules were obtained by aligning RNF146 WWE bound to iso-ADP complex (Protein Data Bank: 3V3L; Wang *et al.* G&D 2012) to each of the two WWE domains of DTX2. Figures displaying molecular structures were prepared with PyMOL (http://www.pymol.org/). For the analysis of DTX2 homodimerization, Colabfold v1.5.2 [https://github.com/sokrypton/ColabFold] along with Alphafold 2.3.1 [https://github.com/google-deepmind/alphafold] were installed and run on the Narval high performance computing cluster provided by the Digital Research Alliance of Canada using NVidia A100SXM4 (40 GB) GPU computing nodes. ColabFold_batch was executed with the following parameters: --use-gpu-relax --amber --num-relax 3 --num-models 3 --templates --num-recycle 30 --recycle-early-stop-tolerance 0.5 --model-type auto. All predicted structures were post analyzed using the AF2multimer-analysis [https://github.com/walterlab-HMS/AF2multimer-analysis]. All interface and contact information were extracted directly from the AF2 multimer-analysis reports.

### Generation of DTX2 KO cell lines

U2-OS cells were transfected with two different DTX2 sgRNAs CRISPR/Cas9 All-in-One Lentivector using JetPRIME (Polyplus) transfection reagent (DTX2 sgRNA1: CTCGATGAAGCTGCAGACGG, DTX2 sgRNA2: GGCTCCCAAGCCCAAAACGT). Puromycin selection was applied 24 h post transfection during 60 h, and cells were then released in media devoid of puromycin. Surviving cells were seeded at low density to form single cell colonies. The lack of DTX2 expression was confirmed by immunoblotting. Genomic DNA was then extracted from KO DTX2 clones, and the CRISPR-targeted region was amplified by PCR. PCR products were sequenced and analyzed using the CRISP-ID website to validate the effective inactivation of all DTX2 alleles (113).

### Streptavidin-mediated proteins pull-downs

Streptavidin pull-downs were performed as described previously (114). Constructs tagged with S-tag, FLAG and streptavidin-binding peptide (pDEST SFB vector was a kind gift from Dr Junjie Chen (MD Anderson Cancer Center, Houston)) were transfected using polyethylenimine into HEK293T cells. Subsequently, 24 h post transfection, cells were treated with 10 μM olaparib or dimethyl sulfoxide for 18 h and then exposed to 2 mM H_2_O_2_ for 10 min. Cells were then washed in ice-cold PBS and flash frozen in liquid nitrogen. Cell lysis was performed in ice-cold lysis buffer 50 mM Tris–HCl pH 7.5, 150 mM NaCl, 1 mM EDTA, 1% NP-40, 10% glycerol, 0.5 mM DTT, 1x protease inhibitor cocktail (Roche), 1 mM NaF, 1 mM Na_3_VO_4_, 1 mM PMSF, supplemented with 1 μM PARG inhibitor (PDD00017273, Sigma-Aldrich) and olaparib (AZD2281, Selleckchem) for 15 min at 4 °C on a rotator. Cell extracts were sonicated 3 times for 10 s at 30% intensity on a Branson Sonicator (Branson 450 Digital Sonifier). After 15 min of centrifugation at 14,000*g* at 4 °C, MagReSyn Streptavidin (Resyn Biosciences) beads were added by pull-down in the supernatant. A portion of the supernatants was also conserved for input controls. Beads were incubated 1 h at 4 °C to capture S protein-FLAG-streptavidin binding peptide (SFB)-tagged proteins. Captured proteins were washed twice with lysis buffer, and the final wash was supplemented with 200 mM NaCl and 1 mM DTT. Beads were resuspended in Laemmli buffer (120 mM Tris pH 6.8, 12% glycerol, 3.67% SDS, 200 mM DTT, and bromophenol blue), heated 5 min at 95 °C and analyzed by immunoblotting. Samples were separated by SDS-PAGE and transferred to polyvinylidene fluoride membranes (Millipore). Detection was done using the specified antibodies.

### Homologous recombination and NHEJ assays

U-2 OS DR-GFP and EJ5-GFP cells were transfected with control or BRCA1, RAD51, FAM35A/SHLD2, and DTX2-targeting siRNAs. Twenty-four hours later, plasmids expressing the I-Sce-I nuclease and in the case of the DR-GFP assays, mCherry were transfected. Subsequently, 48 h later, the efficiency of homologous recombination was evaluated as the % of mCherry-positive cells that were also GFP-positive using a BD FACS Jazz Cell Sorter (BD Biosciences). NHEJ efficiency was evaluated as the % of GFP-positive cells and normalized to that of Ctl siRNA-transfected cells.

### Colony formation assays

U-2 OS or HeLa cells were seeded at low densities in 6-well plates (1000 cells/well for U-2 OS; 500 cells/well for HeLa cells) and cultured for 12 days. In experiments involving siRNA transfection, cells were treated with genotoxic agents 48 h posttransfection. For treatments with olaparib (Selleck Chemicals), cells were incubated with the indicated concentrations throughout the assay period. X-ray irradiation was performed using an X-RAD 225 XL irradiator (Precision X-ray). Following 12 days of incubation, colonies were fixed in 70% ethanol at 4 °C for a minimum of 20 min. Subsequently, colonies were stained with 1% crystal violet in 20% ethanol for 15 min, washed with water, and air-dried. Viable colonies were manually counted for each experimental condition. The surviving fraction at each dose was determined after normalizing to the plating efficiency of untreated samples.

### CellTiter Glo cell viability assay

CellTiter-Glo luminescent cell viability assay (Promega) was used in accordance with the manufacturer’s protocol. RPE1-hTERT Δp53 cells were transfected with control, BRCA1, and DTX2-targeting siRNAs. Twenty-four hours later, cells were seeded in duplicate into 96-well solid white plates (Greiner Bio-One) at a density of 1000 cell/well. Twenty-four hours later, the cells were treated with olaparib and incubated for 5 days with the indicated concentrations. Afterward, a volume of CellTiter-Glo Reagent equal to the volume of cell culture medium present in each well was added and mixed for 2 min on an orbital shaker to induce cell lysis. The plate was then incubated at RT for 10 min to stabilize luminescent signal prior to measurement using a Biotek Synergy HT microplate reader.

### Cell cycle distribution analysis

U-2 OS cells were reverse transfected with siRNAs and 48 h later, 10 μM EdU was added to the media for 30 min. Cells were then washed twice with PBS, trypsinized and collected in 15 ml tubes with complete media. All centrifugation steps were done at 4 °C and 400*g* for 3 min until fixation. After a cold-PBS wash, cells were extracted with CSK buffer (25 mM Hepes (pH 7.4), 50 mM NaCl, 1 mM EDTA, 3 mM MgCl_2_, 300 mM sucrose, 0.5% Triton X-100,protease inhibitor cocktail tablet) for 5 to 10 min on ice. Ice cold-PBS containing 1 mg/ml bovine serum albumin was then added. Cells were fixed with PBS-paraformaldehyde 2% at RT for 20 to 30 min and washed once with BD Perm/wash buffer. Cells were resuspended in freezing buffer (FBS: 10% dimethyl sulfoxide) and stored at −80 °C prior to analysis. Before staining, cells were washed once with storage buffer (PBS, 3% FBS, 0.09% sodium azide) and once with BD Perm/wash. Click chemistry reactions were performed using PBS supplemented with 2 mM CuSO_4_, 2 mg/ml Sodium L-ascorbate and 1 μM Alexa fluor 645 azide, for 30 min at RT. Finally, cells were washed once with BD Perm/wash and resuspended in analysis buffer (0.02% sodium azide, 250 μg/ml RNAse A and 20 μg/ml propidium iodide). All samples were run on a BD Accuri C6 Flow Cytometer (BD Biosciences) and analyzed using the Flowjo software (flowjo.com).

## Data availability

All data described herein are contained within the manuscript and supplementary files. This study generated a collection of plasmids and cell lines. All materials will be distributed upon reasonable request after publication.

## Supporting information

This article contains supporting information.

## Conflict of interest

All authors declare no conflict of interest with the contents of this article.
