# Supplementary material for: An E3 ubiquitin ligase localization screen uncovers DTX2 as a novel ADP-ribosylation-dependent regulator of DNA double-strand break repair
Source: J Biol Chem. 2024 Jul 9;300(8):107545. doi: 10.1016/j.jbc.2024.107545 (PMC11345397; doi:10.1016/j.jbc.2024.107545)
Supplement: Supporting Figure S1 [file mmc1.pdf]

Figure S1. A Localization Screen for Novel Human RING/U-box E3 Ubiquitin Ligases Recruited to DNA Damage.

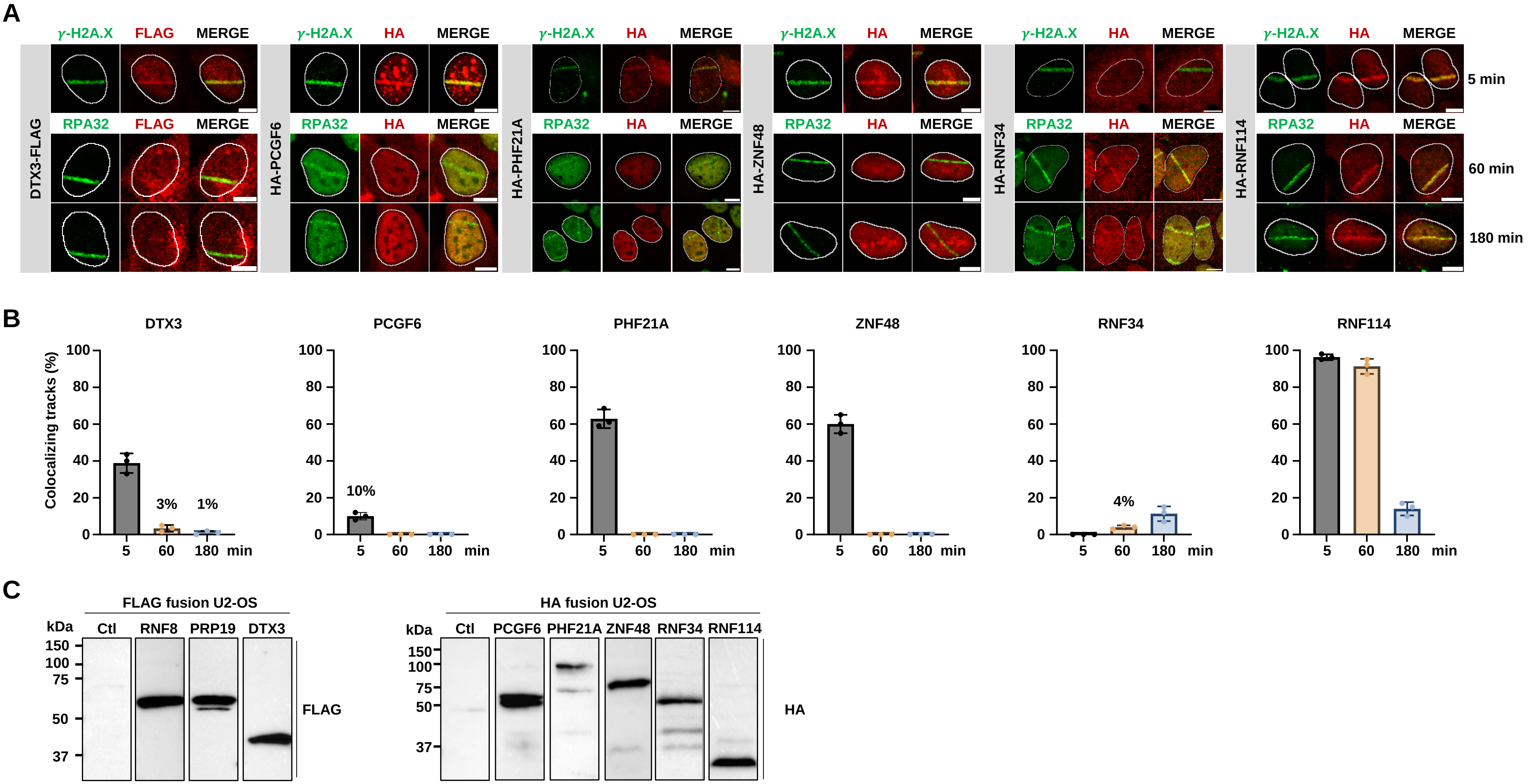

Figure S1. A Localization Screen for Human RING/U-box E3 Ubiquitin Ligases Recruited to DNA Damage. (A) Re-localization of candidate E3 ligases to sites of DNA damage at the indicated post-irradiation times (min). **(B)** Quantification of microirradiated cells with FLAG- or HA-tagged E3 ligases accumulation at γ-H2A.X- and RPA32-positive stripes. Data represent the mean % of cells with E3 ligase/γ-H2A.X or RPA32-colocalizing stripes ± SD (Each data point represent an independent biological replicate (n = 3)). **(C)** Immunoblot validation of FLAG/HA-tagged E3 ligase expression. Scale bar = 10 μm.
