# Supplementary material for: An E3 ubiquitin ligase localization screen uncovers DTX2 as a novel ADP-ribosylation-dependent regulator of DNA double-strand break repair
Source: J Biol Chem. 2024 Jul 9;300(8):107545. doi: 10.1016/j.jbc.2024.107545 (PMC11345397; doi:10.1016/j.jbc.2024.107545)
Supplement: Supporting Figure S2 [file mmc2.pdf]

Figure S2. DTX2 Localizes to Microirradiation Stripes in a PARP-Dependent Manner.

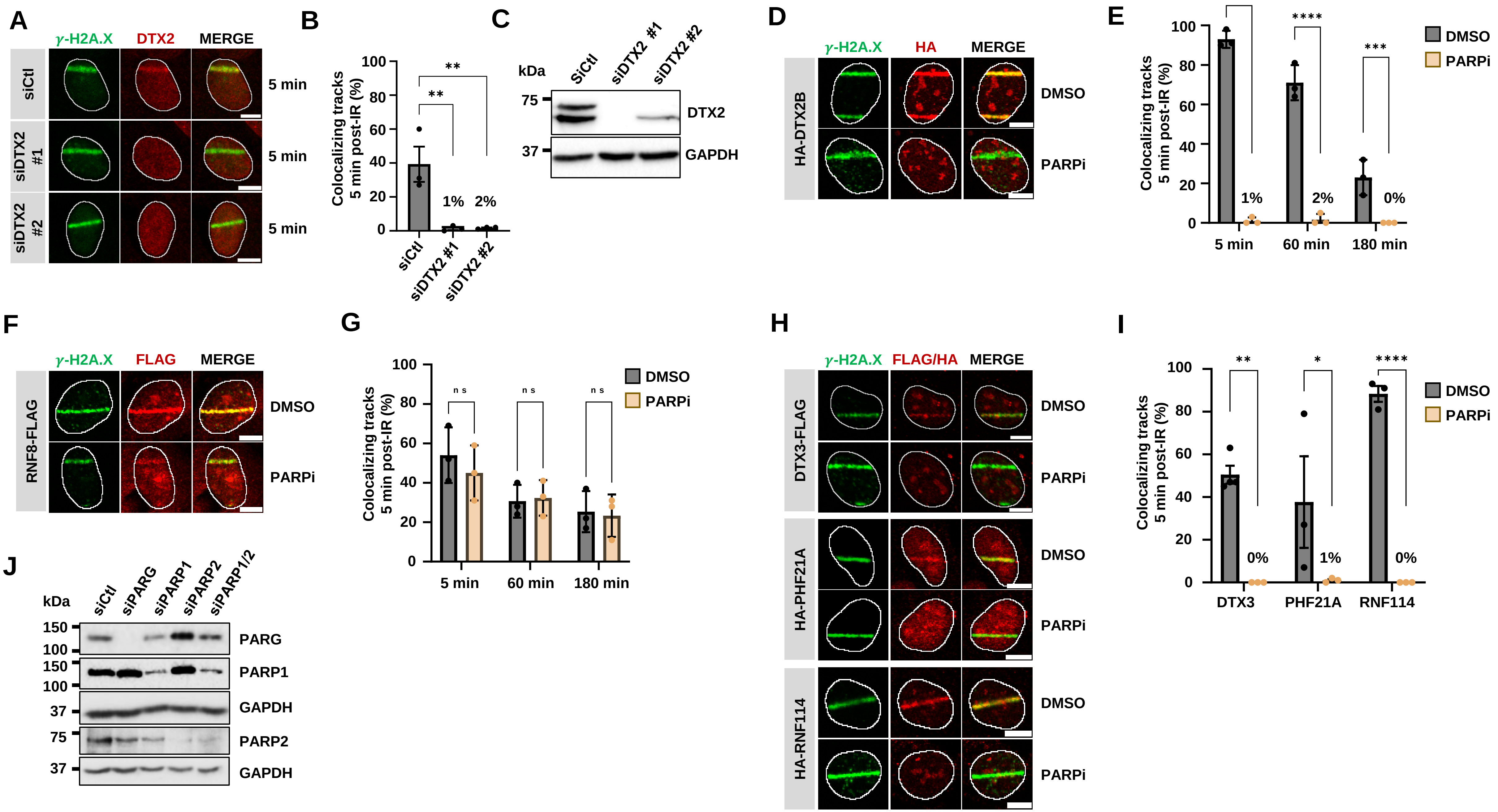

Figure S2. DTX2 Localizes to Microirradiation Stripes in a PARP-Dependent Manner. (A, B, C) Validation of immunofluorescence specificity of the DTX2 antibody. U-2 OS cells were transfected with Ctl or DTX2-targeting siRNAs and microirradiation and IF staining was performed. DTX2 depletion efficiency was confirmed by immunoblotting. Data represent the mean % of cells with endogenous DTX2/g-H2A.X colocalizing stripes ± SEM (n = 3 biological replicates) Statistical significance was established using one-way ANOVA followed by Dunnett's multiple comparison test (P<0.01 (**)**)**. **(D, E, F, G)** U-2 OS cells were individually transduced with lentiviruses encoding HA-tagged DTX2B or FLAG-tagged RNF8 as a control. 48 hrs post-selection, cells were treated with vehicle (DMSO) or 5 mM PARPi (olaparib) for 30 minutes prior to performing microirradiation and IF at the indicated times. **(H, I)** U-2 OS cells expressing the indicated FLAG/HA-tagged E3 ligases were exposed to DMSO or PARPi and microirradiated prior to IF. Data represent the mean % of cells with FLAG/HA/g-H2A.X colocalizing stripes ± SEM (n = 3 biological replicates). Statistical significance was established using Two-Way ANOVA followed by Šídák's multiple comparisons test. (* P<0.05, ** P<0.01, **** P<0.0001**)** **(J)** Immunoblot validation of PARG, PARP1, PARP2 knockdowns. In the bar graphs, each data point represents an independent biological replicate. Scale bar = 10 µm.
