# Supplementary material for: An E3 ubiquitin ligase localization screen uncovers DTX2 as a novel ADP-ribosylation-dependent regulator of DNA double-strand break repair
Source: J Biol Chem. 2024 Jul 9;300(8):107545. doi: 10.1016/j.jbc.2024.107545 (PMC11345397; doi:10.1016/j.jbc.2024.107545)
Supplement: Supporting Figure S3 [file mmc3.pdf]

Figure S3. The WWE1/2 and DTC Domains of DTX2 Mediate its Recruitment to Microirradiation Stripes.

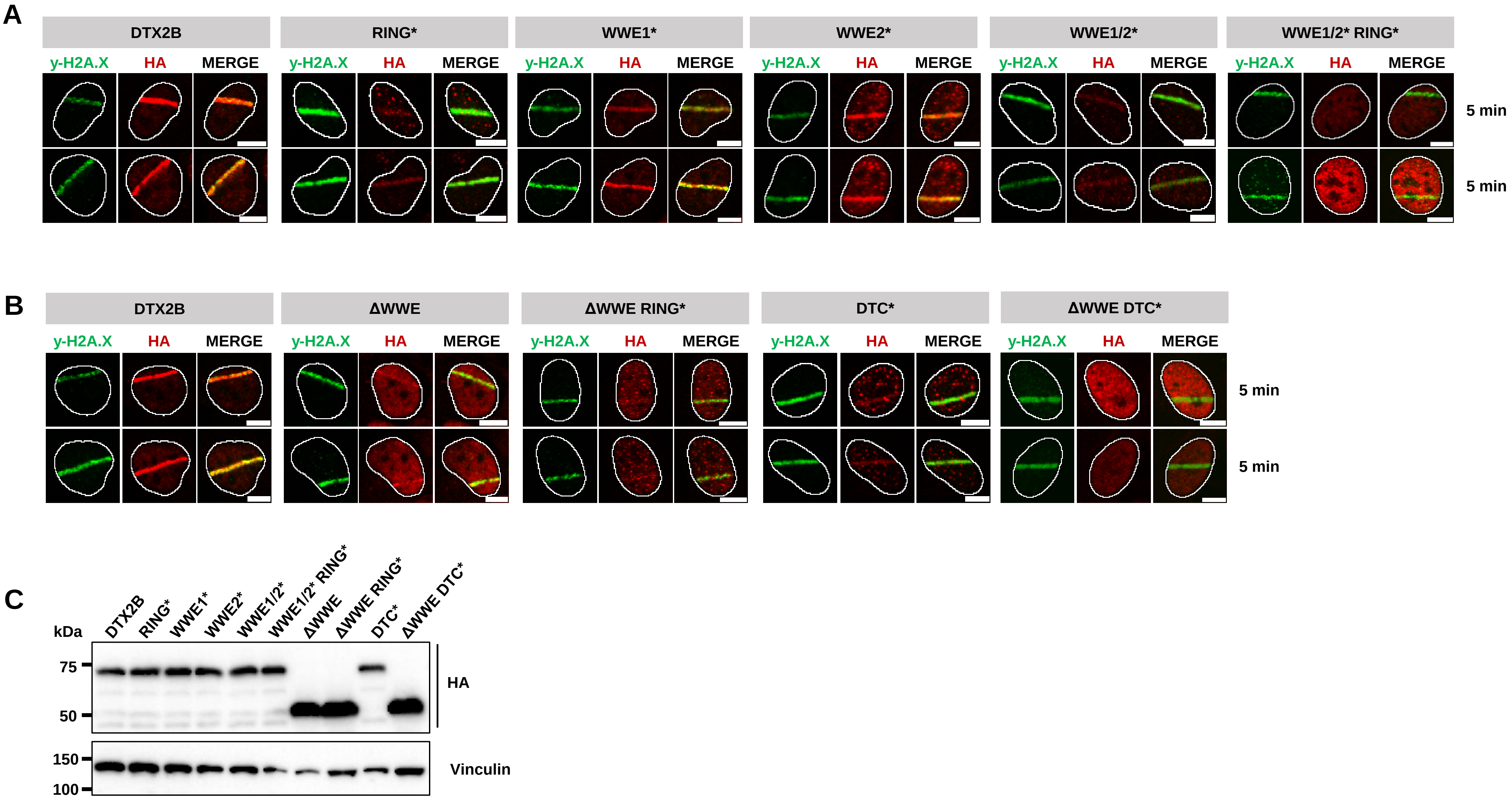

Figure S3. The WWE1/2 and DTC Domains of DTX2 Can Mediate its Recruitment to Microirradiation Stripes. (A, B) Representative images of microirradiated cells transduced with lentiviruses encoding the indicated HA-tagged DTX2 constructs. **(C)** Assessment of DTX2 construct expression by immunoblotting. Scale bar = 10 µm.
