# Supplementary material for: An E3 ubiquitin ligase localization screen uncovers DTX2 as a novel ADP-ribosylation-dependent regulator of DNA double-strand break repair
Source: J Biol Chem. 2024 Jul 9;300(8):107545. doi: 10.1016/j.jbc.2024.107545 (PMC11345397; doi:10.1016/j.jbc.2024.107545)
Supplement: Supporting Figure S4 [file mmc4.pdf]

Figure S4. The WWE domains and DTC domains of DTX2 associate with ADP-ribosylated proteins *in vivo*

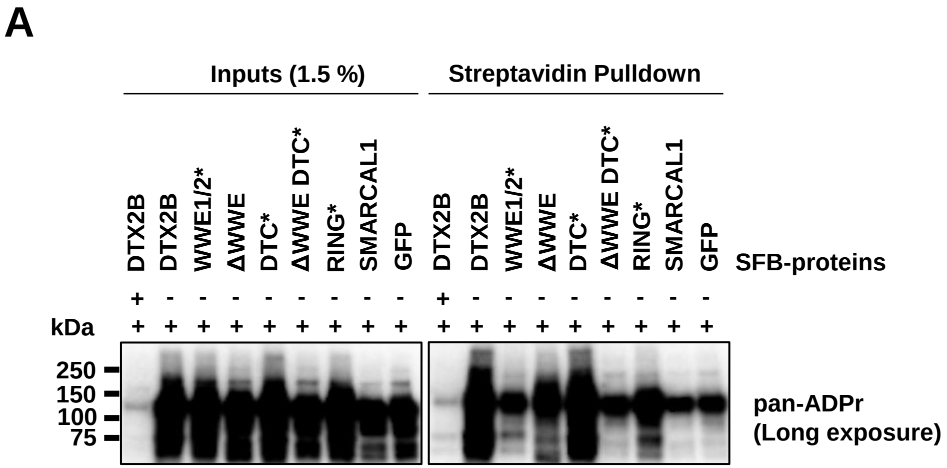

Fig. S4. The WWE domains and DTC domains of DTX2 associate with ADP-ribosylated proteins *in vivo*. HEK293 cells were individually transfected with plasmids encoding SFB-tagged DTX2B constructs or SMARCAL1 and EGFP controls. 48 hrs later, cells were treated with H_2O_2 in the presence or absence of olaparib, lysed and streptavidin pulldown was performed. DTX2B and its interactors were detected by immunoblotting using the indicated antibodies. A long exposure of the pan-ADPr panel from **Fig. 4F** reveals that the WWE1/2* mutant has limited but still detectable interaction with ADP-ribosylated proteins compared with SFB-SMARCAL1 and SFB-GFP controls.
