# Supplementary material for: An E3 ubiquitin ligase localization screen uncovers DTX2 as a novel ADP-ribosylation-dependent regulator of DNA double-strand break repair
Source: J Biol Chem. 2024 Jul 9;300(8):107545. doi: 10.1016/j.jbc.2024.107545 (PMC11345397; doi:10.1016/j.jbc.2024.107545)
Supplement: Supporting Figure S5 [file mmc5.pdf]

Figure S5. DTX2 Influences Double-Strand Break Repair.

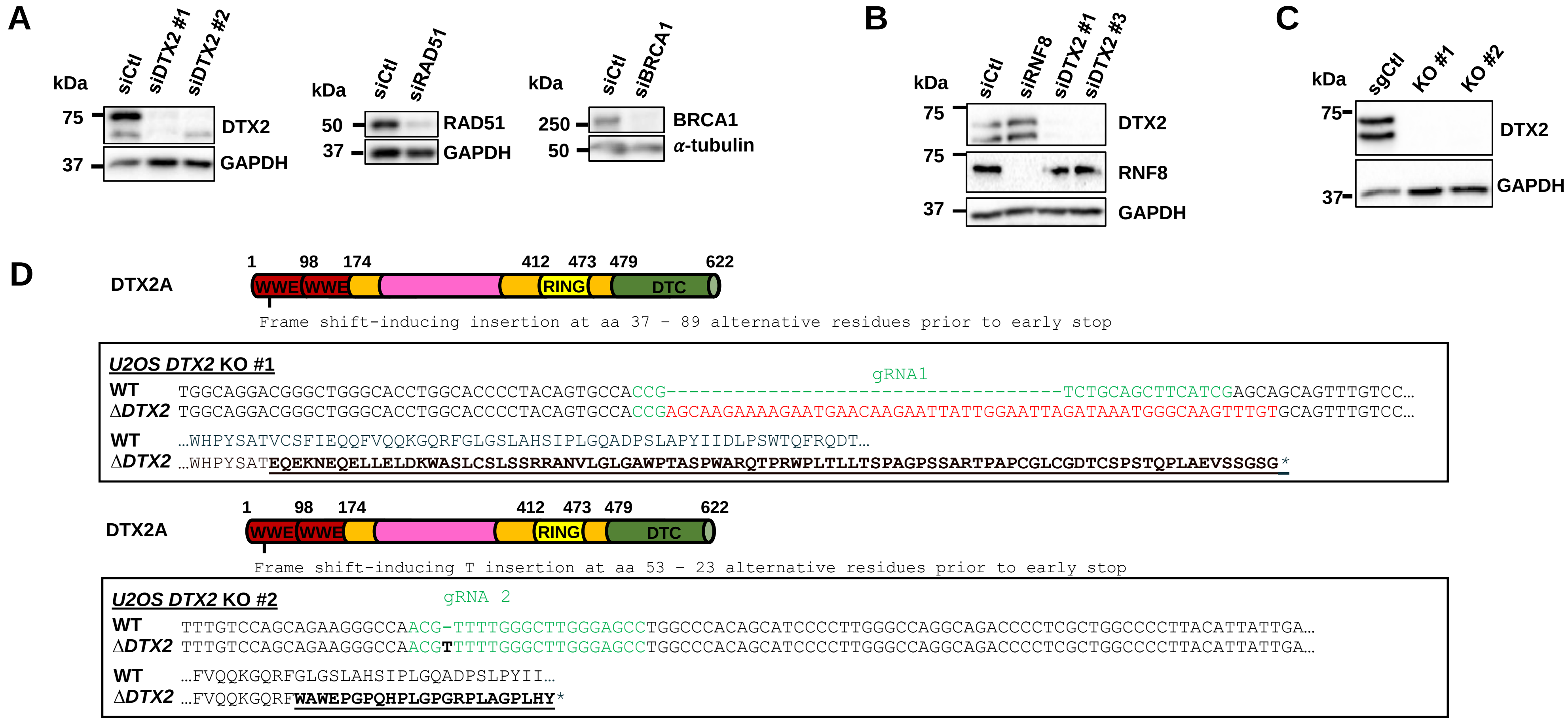

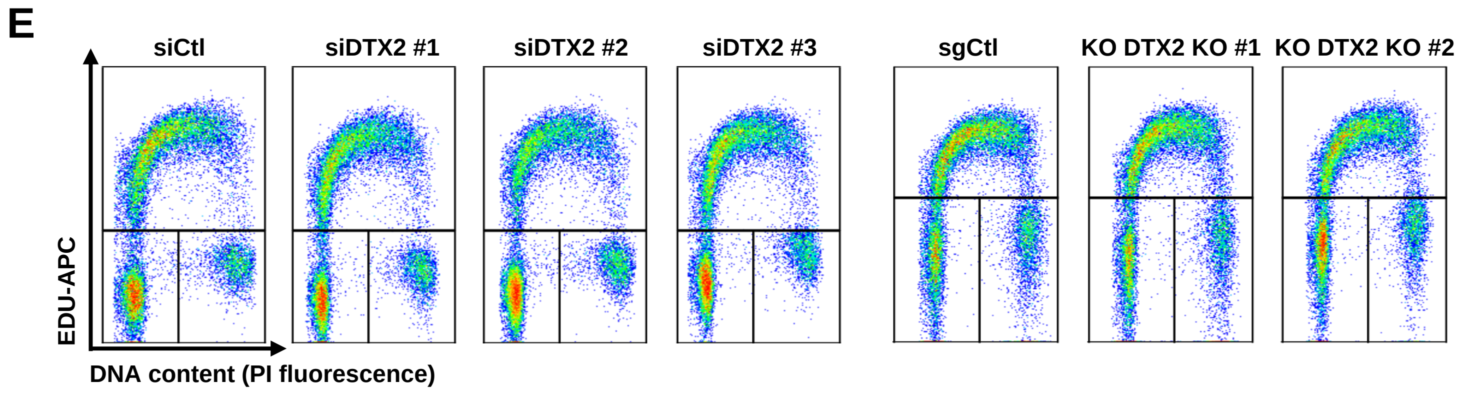

F

Cell Lines	G0/G1-Phase	S-Phase	G2/M-Phase
siCtl	29.4% ± 1.1	54.8% ± 3.3	14.8% ± 3.0
siDTX2 #1	29.9% ± 2.4	55.2% ± 1.3	12.8% ± 0.7
siDTX2 #2	40.1% ± 2.2	43.4% ± 2.1	14.4% ± 2.8
siDTX2 #3	32.6% ± 1.3	55.6% ± 1.4	11.8% ± 0.1
sgCtl	28.7% ± 6.1	54.4% ± 3.2	14.2% ± 3.8
KO #1	29.8% ± 7.4	54.9% ± 4.9	12.2% ± 0.9
KO #2	35.2% ± 8.1	51.2% ± 7.4	12.1% ± 0.4

Figure S5. DTX2 Influences DNA Double-Strand Break Repair Pathway. (A) Immunoblot validation of knockdowns for the DR-GFP and EJ5-GFP assays. **(B, C)** Immunoblot validation of knockdowns for the 53BP1 and BRCA1 foci assays. **(C)** Immunoblot validation of U-2 OS DTX2 KO cell lines. **(D)** DTX2 KOs were validated by PCR amplification of the targeted region followed by CRISP-ID Analysis. **(E, F)** Cell cycle distribution was determined by EdU incorporation and PI labeling 48 h post-transfection of U-2 OS cells with the indicated siRNAs. The FACS profiles are representative of experiments performed in biological triplicates, Experiments were performed in biological triplicates. The table lists the average % of cells in each cell cycle phase -/+ standard deviation. Cell cycle distribution for control (sgCtl) and DTX2 KO U-2 OS cell lines was performed similarly. Scale bar = 10 µm.
