# Supplementary material for: An E3 ubiquitin ligase localization screen uncovers DTX2 as a novel ADP-ribosylation-dependent regulator of DNA double-strand break repair
Source: J Biol Chem. 2024 Jul 9;300(8):107545. doi: 10.1016/j.jbc.2024.107545 (PMC11345397; doi:10.1016/j.jbc.2024.107545)
Supplement: Supporting Figure S6 [file mmc6.pdf]

Figure S6. DTX2 Promotes Resistance to X-ray Irradiation and Olaparib

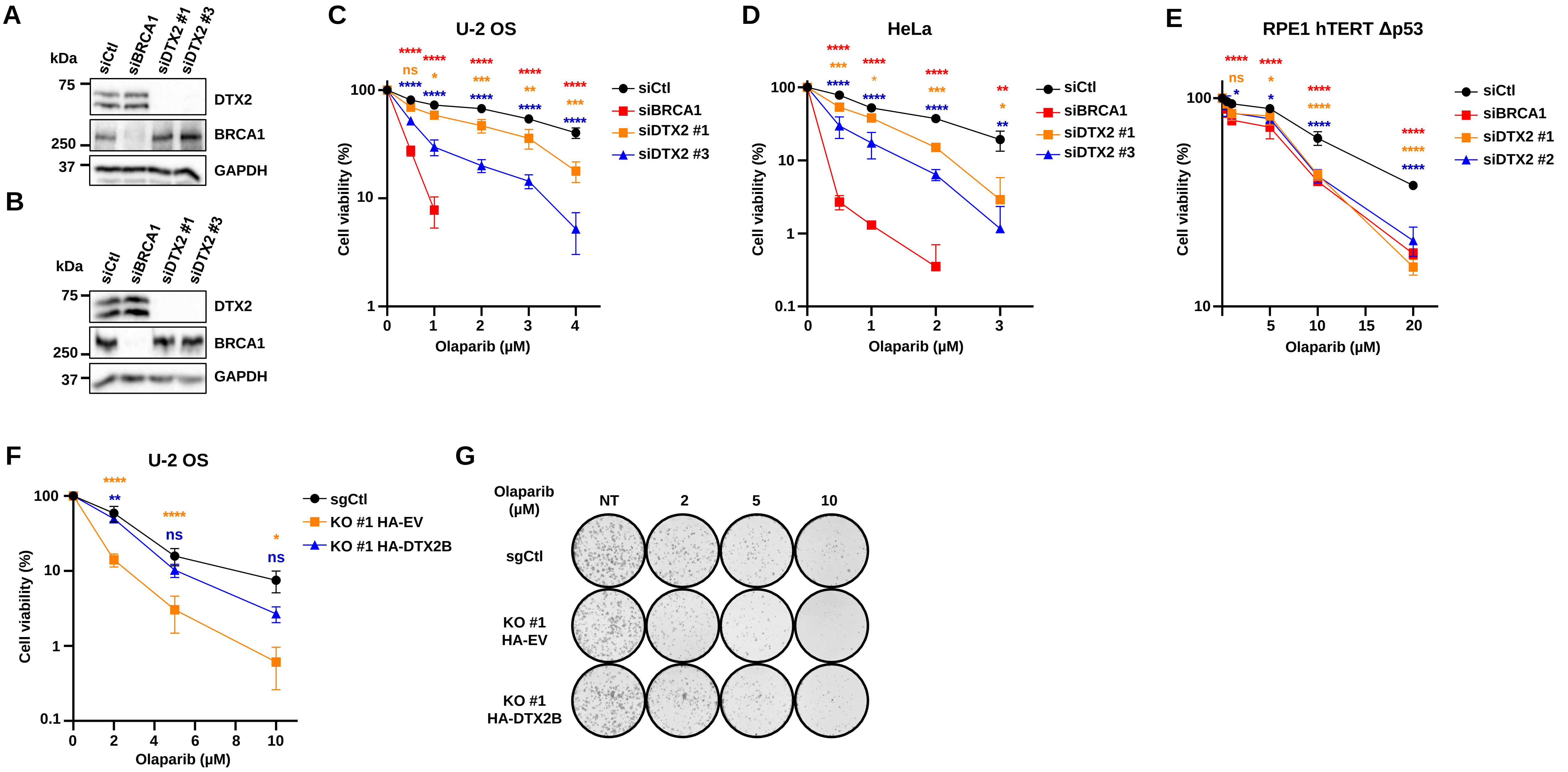

Figure S6. DTX2 Promotes Resistance to X-ray Irradiation and Olaparib. (A, B) Immunoblot validation of knockdowns in support of U-2 OS and HeLa cells X-ray irradiation colony formation assays **(C, D)** U-2 OS or HeLa cells were transfected with the indicated siRNAs and 48 h later exposed to the indicated doses of olaparib. Colony formation assays were performed over 12 days. **(E)** RPE1 Dp53 cells were transfected with the indicated siRNAs and 48 h later treated with the indicated doses of olaparib, cell viability was assessed by CellTiter Glo 5 days later. **(F, G)** sgCtl or DTX2 KO cells stably transduced with empty vector (EV) or HA-DTX2B were exposed to the indicated olaparib doses and colony formation assays were done over 12 days. For all experiments, ≥ 3 biological replicates were done, and data represents the mean -/+ standard error of the mean. Statistical significance was computed using 2-way ANOVA with Tukey's multiple comparisons (* $P<0.05$, *** $P<0.001$, **** $P<0.0001$).
