# Supplementary material for: An E3 ubiquitin ligase localization screen uncovers DTX2 as a novel ADP-ribosylation-dependent regulator of DNA double-strand break repair
Source: J Biol Chem. 2024 Jul 9;300(8):107545. doi: 10.1016/j.jbc.2024.107545 (PMC11345397; doi:10.1016/j.jbc.2024.107545)
Supplement: Supporting Figure S7 [file mmc7.pdf]

Figure S7. RNF114 is recruited to DNA lesions via its Zinc Fingers and UIM.

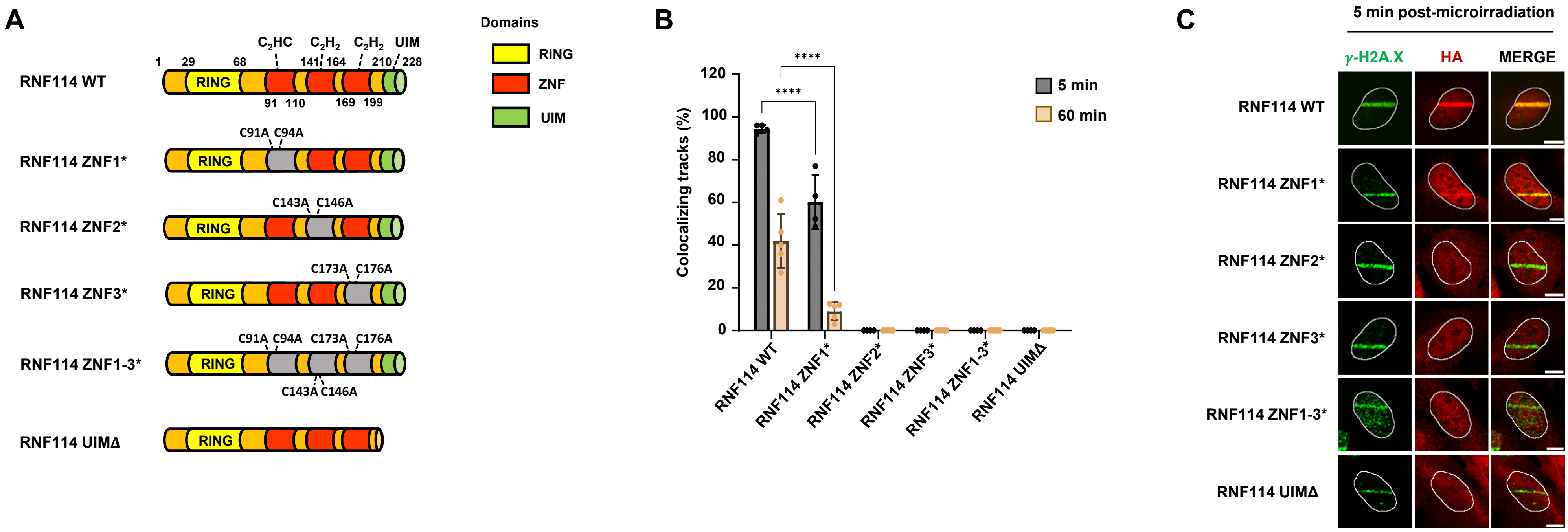

Figure S7. RNF114 is Recruited to Microirradiation Sites Via its Zinc Fingers and its Ubiquitin-Interacting Motif. (A) Schematic representation of RNF114 WT and mutant constructs **(B, C)** U-2 OS cells were individually transduced with lentiviruses encoding HA-tagged WT RNF114 or the indicated mutants. 48 hrs post-selection, cells were microirradiated and immunofluorescence staining for HA and γ-H2A.X as early (5 min) or RPA32 as late (60 min) DNA damage markers. Statistical significance was computed using 2-way ANOVA followed by Šídák's multiple comparisons test (**** $P<0.0001$). In the bar graphs, each data point represents an independent biological replicate. Scale bar = 10 μm.
