# Supplementary material for: An E3 ubiquitin ligase localization screen uncovers DTX2 as a novel ADP-ribosylation-dependent regulator of DNA double-strand break repair
Source: J Biol Chem. 2024 Jul 9;300(8):107545. doi: 10.1016/j.jbc.2024.107545 (PMC11345397; doi:10.1016/j.jbc.2024.107545)
Supplement: Supporting Figure S8 [file mmc8.pdf]

Figure S8. DTX2 may homodimerize via its RING-DTC module.

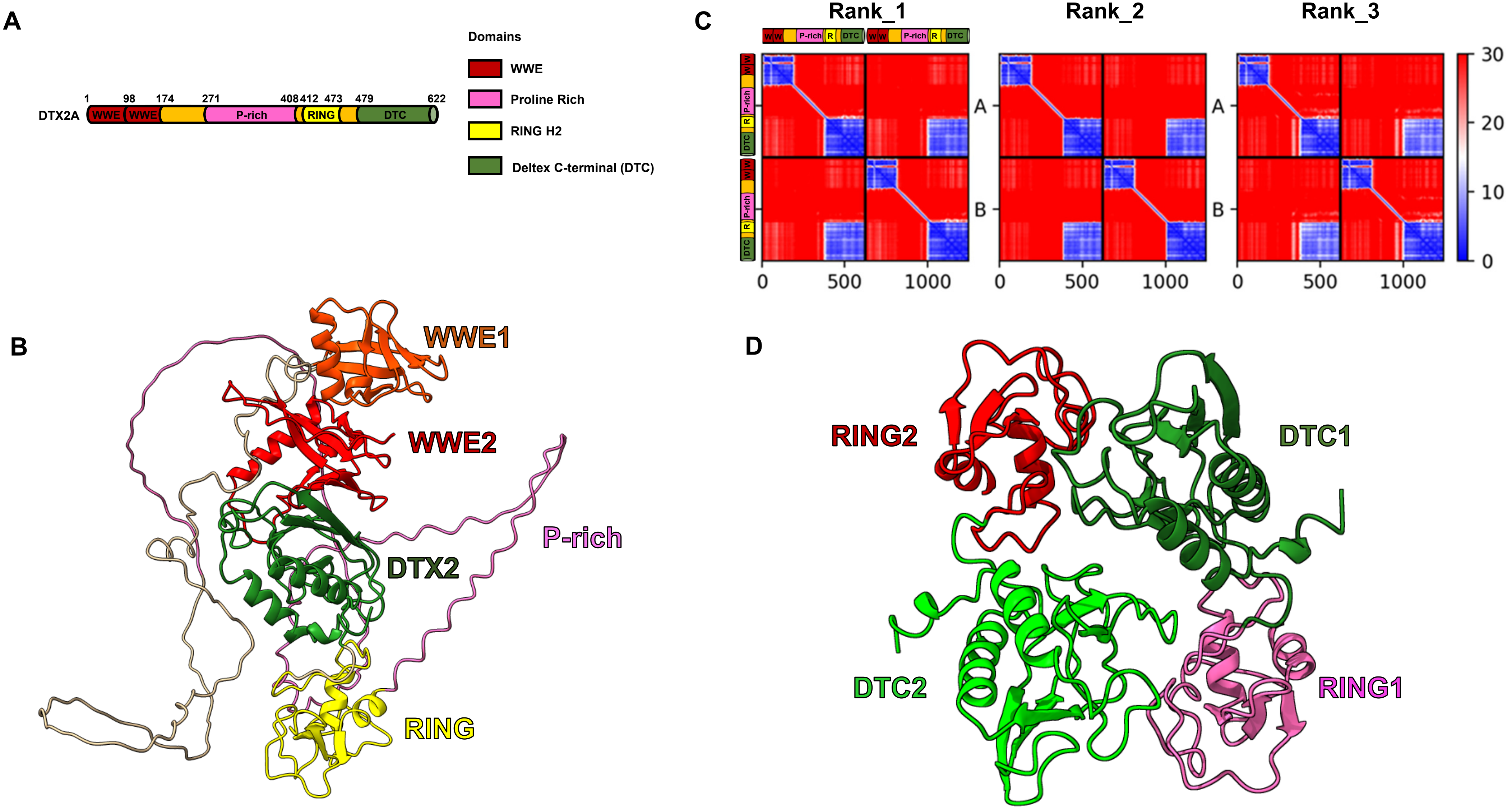

Fig. S8. DTX2 may homodimerize via its RING-DTC module. (**A**) Schematic representation of DTX2A domain organization. (**B**) Predicted structure of monomeric full length DTX2A generated by AlphaFold. (W: WWE domains, P-rich, R: RING, DTC: Deltex C-terminal (**C**) Predicted alignement error plots of DTX2 dimers obtained from AlphaFold2 multimer predictions. (**D**) Structure predictions of DTX2A homodimer organization. The RING domains are depicted in pink/red for DTX2 monomers 1 and 2 respectively. DTC domains are depicted in WWE domains and unstructured regions were left out for clarity purposes. Colabfold summary output and predicted inter-monomer interfaces that occur between RING1/DTC2 (pink/lime) and RING2/DTC1 (red/green) and are presented in **Table S4**.
