# Supplementary material for: An E3 ubiquitin ligase localization screen uncovers DTX2 as a novel ADP-ribosylation-dependent regulator of DNA double-strand break repair
Source: J Biol Chem. 2024 Jul 9;300(8):107545. doi: 10.1016/j.jbc.2024.107545 (PMC11345397; doi:10.1016/j.jbc.2024.107545)
Supplement: Supporting Figure S9 [file mmc9.pdf]

Figure S9. Multiple DELTEX Ubiquitin Ligases are Recruited to Microirradiation Stripes.

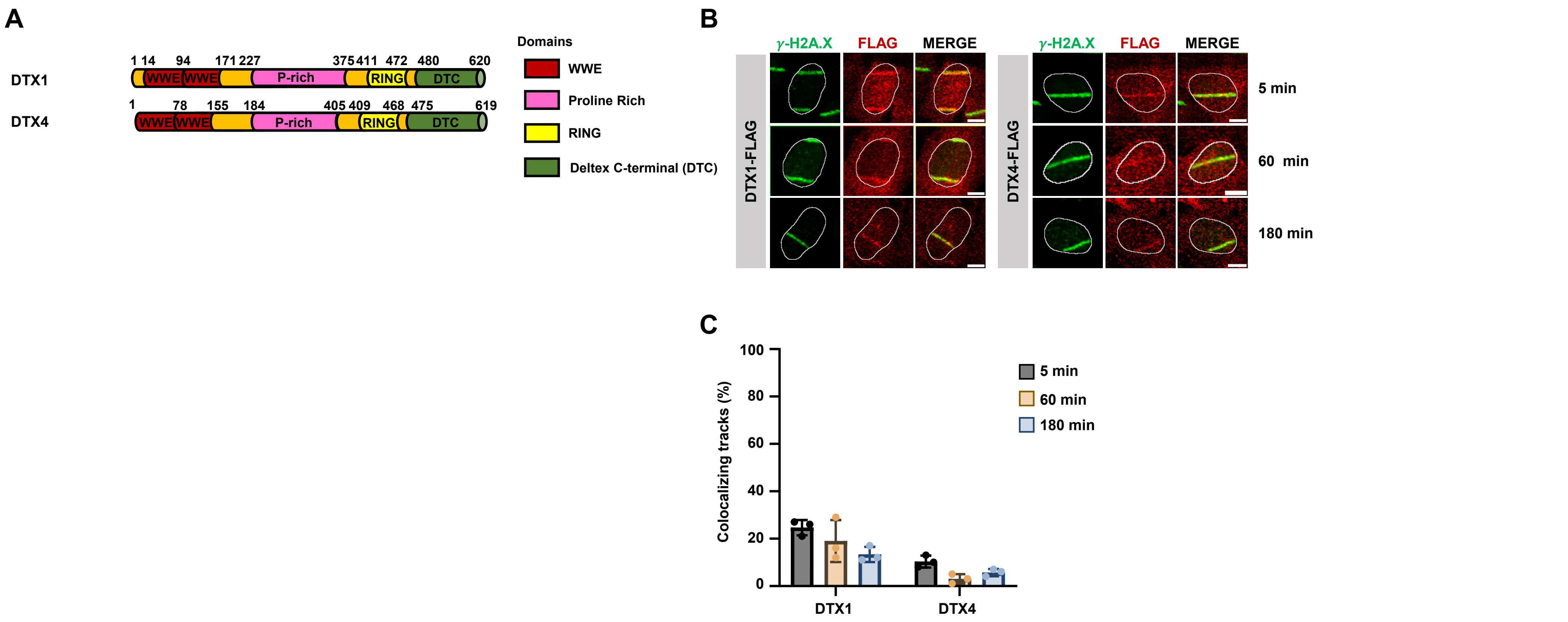

Figure S9. Multiple DELTEX Ubiquitin Ligases are Recruited to Microirradiation Stripes. (A) Schematic representation of DELTEX E3 ligases tested for localization **(B, C)** U-2 OS cells were individually transduced with lentiviruses encoding FLAG-tagged DTX1 and DTX4 proteins. 48 h post-selection, cells were microirradiated and immunofluorescence staining for FLAG and γ-H2A.X as a DNA damage marker. Data represent the mean % of cells with colocalizing FLAG/γ-H2A.X ± SD (data points in the bar graph represent biological replicates (n=3)). Scale bar = 10 μm.
